# Understanding the Difficulties in Diagnosing Neonatal Sepsis: Assessing the Role of Sepsis Biomarkers

**DOI:** 10.2478/jccm-2024-0039

**Published:** 2024-10-31

**Authors:** Nicoleta Lungu, Ana-Maria-Cristina Jura, Daniela-Eugenia Popescu, Florin George Horhat, Aniko Maria Manea, Marioara Boia

**Affiliations:** Department of Obstetrics and Gynecology, “Victor Babes” University of Medicine and Pharmacy, Timișoara, Romania; University of Medicine and Pharmacy Victor Babes Timisoara, Timisoara, Romania; Multidisciplinary Research Center on Antimicrobial Resistance (MULTI-REZ), Microbiology Department, “Victor Babes” University of Medicine and Pharmacy, Timișoara, Romania

**Keywords:** neonatal sepsis, biomarkers, ferritin, lactate dehydrogenase, neonatal intensive care unit

## Abstract

**Background:**

Neonatal sepsis is a serious condition with high rates of morbidity and mortality, caused by the rapid growth of microorganisms that trigger a systemic reaction. Symptoms can range from mild to severe presentations. The causative microorganism is usually transmitted from mothers, especially from the urogenital tract, or can originate from the community or hospital.

**Methods:**

Our retrospective study assessed 121 newborns, including both preterm and term infants, divided into three groups within the first 28 days of life: early-onset sepsis (35), late-onset sepsis (39), and a control group (47). Blood samples and cultures were obtained upon admission or at the onset of sepsis (at 24 and 72 hours). The study aimed to evaluate the limitations of commonly used biomarkers and new markers such as lactate dehydrogenase and ferritin in more accurately diagnosing neonatal sepsis.

**Results:**

Our study revealed a significant difference between the initial and final measures of lactate dehydrogenase (LDH) and ferritin in the early-onset sepsis (EOS) and late-onset sepsis (LOS) groups.

**Conclusion:**

Ferritin and LDH may serve as potential markers associated with systemic response and sepsis in cases of both early and late-onset sepsis. Monitoring these biomarkers can aid in the timely detection and management of sepsis, potentially improving patient outcomes.

## Introduction

Newborn sepsis, a serious condition caused by bacterial systemic infection, is known to have severe clinical manifestations and can even lead to death [1]. Moreover, it has been linked to long-term impairments that are irreversible and have a signific and impact on the health and development of newborns [2]. Early onset sepsis (EOS) occurs in approximately 1–2‰ of all live births [3]. When referring to EOS, it is important to note that this term encompasses infections occurring within the first 3 days of life (less than 72 hours), although some studies extend the timeframe to include the first week of life [4,5,6,7,8] for patients who require continuous hospitalization. The underdeveloped immune system of premature infants makes them particularly susceptible to infections, triggering an inflammatory response that can lead to atypical clinical-biological manifestations [9]. Shah et al. demonstrated a correlation between gestational age and sepsis frequency - 33% of infants before 28 weeks, increasing to 60% before 25 weeks [6]. Medical advances have improved survival for extremely premature, low birth weight infants [9]. Late onset sepsis (LOS), occurs after the fourth or seventh day of life [10,11] during the neonatal period [12,13]. Cell hypoxia induces a switch from aerobic metabolism to the less efficient anaerobic metabolism in which pyruvate dehydrogenase (PDH) is inhibited and lactate formation from pyruvate by lactate dehydrogenase (LDH) is favored [14]. Ferritin is a key molecule that serves to reduce pro-oxidant stress commonly found in inflammatory conditions [15]. Ferritin is also a member of the protein family that conducts the cellular defense against stress and inflammation, not only as an iron regulatory protein [16].

The gold standard for diagnosing LOS is blood or urine cultures, but these methods have low sensitivity and are time consuming [7,15]. Therefore, healthcare professionals should be aware of the limitations when using these techniques to identify and confirm LOS cases.

The most commonly used biomarkers, such as procalcitonin (PCT), full blood count and C-Reactive Protein (CRP), have limited sensitivity, specificity, and positive predictive value due to the lack of specific clinical signs associated with the condition [17]. To assess organ dysfunction and severity accurately, validated scoring systems are utilized [18]. The ideal biomarker for sepsis should exhibit high sensitivity and specificity to prevent the overlooking of cases and unnecessary neonatal treatments. It should also be easily assessable, allowing for quick and efficient diagnosis [19]. However, it is of utmost importance for a biomarker to exhibit a high level of accuracy in promptly detecting the presence or absence of neonatal sepsis, as this plays a pivotal role in ensuring efficient treatment and favorable outcomes for infants [20]. Consequently, broad-spectrum antibiotics that have been empirically selected are frequently administered to high-risk infants, based on the suspicion of sepsis [1]. Delaying the initiation of treatment in a newborn with suspected sepsis, not only hampers the chances of recovery but also significantly increases the likelihood of the disease progressing unfavorably [21]. Therefore, the exploration of alternative strategies, such as the utilization of biomarkers, becomes paramount in our quest to address this formidable challenge [22]. Lactate dehydrogenase, hemoglobin, and ferritin show promising results as markers for sepsis [23].

The aim of this study is to thoroughly examine and establish that the biomarkers and laboratory findings currently employed for diagnosing neonatal sepsis, whether it occurs early or late in after birth, may possess notable constraints. Our objective is to enhance the accuracy and reliability of early sepsis diagnosis by including and assessing additional biomarkers such as lactate dehydrogenase (LDH) and ferritin, in addition to conventional markers like C-reactive protein (CRP) and procalcitonin (PCT). This comprehensive approach aims to emphasize the deficiencies of current diagnostic methods and suggest more efficient alternatives, ultimately enhancing the diagnosis and treatment of neonatal sepsis to improve patient outcomes.

## Materials and Methods

### Ethics and Design of the study

This retrospective study assesses newborns who were admitted to the Neonatal Intensive Care Unit of the “Louis Țurcanu” Children Emergency Hospital from Timișoara over a 2-year period. The focus of the study was on newborns with sepsis or clinical signs indicating sepsis. Ethical approval of the study has been obtained from the Research Ethics Committee of the University of Medicine and Pharmacy “Victor Babeș” of Timișoara, CECS Opinion no. 57/2018 and from the Ethics Committee of the “Louis Țurcanu” Children Emergency Hospital Timișoara no.12/24.03.2022. Furthermore, written informed consent has been obtained from the parents of the newborns who were included in the study.

### Inclusion Criteria and Study Variables

The study included a total of 121 newborns, who were divided into three distinct populations based on the occurrence of sepsis. These populations include: early onset sepsis, late onset sepsis and a control group consisting of individuals with similar characteristics to the study groups, but with sepsis that was culture proven.

Both preterm and term newborns were enlisted for participation in the study, and they were recruited from the Department of Neonatology of “Louis Țurcanu” Children Emergency Hospital. The recruitment period for this study spanned from the first of January 2022 and 31^st^ of December 2023.

The selection process of those newborns was carried out meticulously, as they were chosen based on specific criteria for inclusion and exclusion. The study included patients born in level I or II maternities and transferred into our clinic within the first few hours after birth. It is important to highlight that our clinic does not possess a maternity department, but rather a neonatal department, as all admitted patients are born in other clinics and transferred if necessary.

The inclusion criteria established for this study were as follows: premature newborns with a gestational age ranging from 24 to 37 weeks as well as term newborns who were less than 28 days old. However, certain cases were excluded from the study. Specifically, neonates with congenital heart disease, neurological abnormalities, and renal malformations were not included in the final analysis.

### Demographic and exploratory data

The demographic information was obtained from the clinical records of patients admitted to our NICU. This study was conducted as an extension of a previously published study regarding hemoglobin, ferritin and lactate dehydrogenase as biomarkers for sepsis in the same NICU and conducted by the same first author. The present study compares the performance of C-reactive protein, procalcitonin, leukocyte count, platelets, neutrophils, ferritin, and lactate dehydrogenase as bio-markers in both EOS and LOS, highlighting differences in biomarker levels at various time points post-infection. Furthermore, it provides a comparative analysis of multiple biomarkers, suggesting that while ferritin and LDH are effective, combining these with other markers like CRP and procalcitonin can enhance diagnostic accuracy. It also discusses the role of these biomarkers in monitoring disease progression and prognosis.

### Study Population, Description and Period

All newborns with clinical indications of sepsis were included in the study. Only the initial episode of sepsis was considered for individuals who have been hospitalized for an extended period and experienced multiple instances of systemic infection. In several cases of late-onset sepsis (LOS), the infection developed after the patients underwent surgical interventions, specifically occurring more than 72 hours post-surgery. This time frame indicates that the sepsis was not present immediately following the surgical procedures but emerged as a complication within the critical period beyond the initial 72 hours. Moreover, there were oxygen supplementation in different forms, from free-flow oxygen to mechanical ventilation.

### Laboratory Procedures

Blood samples were collected at admission or at the onset of sepsis, and then again at 24 and 72 hours after the onset of sepsis, respectively. We gathered samples for hematology, biochemistry, immunology analysis, as well as peripheral and central culture assessment. Also, it is our clinic’s protocol that for all neonates admitted, the following laboratory tests should be performed: complete blood count, c-reactive protein, procalcitonin, ferritin, lactate dehydrogenase,

The blood sample for the complete blood count was collected in small vacutainers with purple stopper with 0.5ml EDTA and were then analyzed in the laboratory using either the SYSMEX XN 1000 or SYSMEX XN-550 automatic hematology analyzer.

The diagnostic thresholds for sepsis involving white blood cells (WBC), typically falls above 30,000/mm^3^ or below 5000/mm^3^ [24]. For platelets, we deemed values below 150.000/mm^3^ to be relevant.

C- reactive protein (CRP) is synthesized by the liver and serves as an inflammatory response to different insult agents. CRP levels show an initial surge within the first 12 hours after infection, remain elevated for a few hours and gradually decline thereafter [3].

Ferritin is being considered for its potential use in rapidly diagnosing neonatal sepsis [25]. We evaluated values exceeding 200ng/ml as positive.

Lactate dehydrogenase (LDH) is an intracellular enzyme that is responsive to energy deficits in all organs and also serves as an indicator of tissue hypoxia [26]. We considered levels above 600U/l to be positive.

Procalcitonin (PCT) is produced in the C cells of the thyroid gland and acts as a precursor to calcitonin. Its levels rise quickly after exposure to endotoxins, particularly bacterial ones, peaking at 6-8 hours and staying high for 48 hours after bacterial threat has passed [3]. The threshold for diagnosing neonatal sepsis is a PCT level greater than 1ng/ml. During neonatal sepsis, PCT is significantly produced in the liver, leading to a potential increase in plasma concentrations by up to 1000 times [27]. In some cases, this parameter may rise after birth, reaching peak values at 24 hours of life (mean 1.5–2.5 ng/mL, range 0.1–20 ng/mL), then returning to normal levels by 72 hours [28].

Immunological assays for procalcitonin were conducted using the automatic immunological analyzer COBAS e411.

Biochemistry tubes with red stoppers containing samples for C reactive protein, ferritin, and LDH were analyzed using the COBAS INTEGRA 400 PLUS and COBAS 6000 devices.

Cultures were collected from a peripheral vein, under rigorous aseptic conditions. Peripheral cultures were grown on sterile containers with seeding medium or were sown in the laboratory and incubated on a thermostat using VITEK 5 COMPACT 15 equipment. The blood sample was inoculated into a BacTALERT PF blood culture flask and placed in the BacTALERT within 2 hours of collection. The sample was collected prior to the start of antibiotic treatment.

Wound cultures were collected after surgery, while cultures from central catheters, drainage or intubation probes were obtained using the same equipment as for peripheral cultures. The Clinical Medical Analysis Laboratory of the Emergency Clinical Hospital for Children “Louis Țurcanu” Timișoara is accredited by RENAR for conducting medical analyses in accordance with SR EN ISO 15189:2013.

### Statistical Analysis

An exploratory analysis was conducted to understand the distribution and characteristics of the variables of interest within each population group. Descriptive statistics, including frequency distribution for categorical variables and mean and standard deviation for continuous variables, were used to summarize the data. This analysis helped gain insights into the different population groups and their respective variable distributions and characteristics.

As the numerical variables considered in our study were found not to be normally distributed, differences between the initial and final measures within each group (EOS, LOS, Control), were tested using the Wilcoxon rank sum test. This statistical test evaluated the significance of differences between the first and last measurements, gestation, type of delivery and perinatal history, and provided insights into temporal changes within these populations, as well as the presence of antenatal risk factors. Laboratory tests and cultures were collected for all our patients, and no deaths occurred during the first 72 hours after onset. Additionally, pairwise comparisons were conducted using the same test between the second measurements of numerical variables across EOS, LOS, and Control groups to discern specific differences between these populations at a particular time point.

An a priori power analysis was conducted to ensure the study had sufficient power to detect meaningful differences within and between groups. Using R Studio software, version 2023.09.1+494 for Mac, with the “pwr” package, we calculated the required sample size based on a medium effect size, following Cohen’s guidelines, and the standard deviations obtained from preliminary data. The analysis indicated that to achieve 80% power at a 5% significance level and a Cohen’s effect size of 0.5 for our primary outcome, a sample size of 64 participants per group was required. However, due to the low NICU admission rates, our study included a total of 121 patients, divided into the early-onset sepsis (EOS) group with 35 participants, the late-onset sepsis (LOS) group with 39 participants, and the control group with 47 participants. Despite not meeting the required sample size for each group, the study still reached significant differences, highlighting the robustness of the observed associations and the potential of the biomarkers under investigation.

The chi-squared test was used to examine the relationship between categorical variables. This test evaluated the presence of statistically significant relationships or dependencies among categorical variables within and between the EOS, LOS, and Control groups. The results were analyzed to identify any possible correlations or connections between these variables.

We used Receiver Operating Characteristic (ROC) curves to determine the biomarker with the best diagnostic effectiveness. This methodical approach allowed for a detailed evaluation of each biomarker’s performance through an assessment of their sensitivity and specificity. We used ROC curves to compare sensitivity and specificity at various thresholds. This thorough analysis emphasized the accuracy of each biomarker for diagnosis and identified the one with the best diagnostic capabilities. We conducted a thorough assessment to develop a better biomarker for early sepsis detection, aiming to improve clinical outcomes by intervening promptly. All statistical analyses were performed using ***R-4.3.2*** with a significance level set at α = 0.05.

## Results

The comprehensive and detailed demographic data, including information on delivery mode, the distribution of gender, gestational age, and birth weight, which have been collected from the clinical records of patients, serves as the foundation for our retrospective study. In order to provide a clear overview, Table 1 presents these findings. The sample sizes varied across the three groups: EOS had 35 participants, LOS had 39 participants and the control group had 47 participants.

**Table 1.** Descriptive statistics

**Variables**	**EOS**	**LOS**	**Control**	**Overall**
n=	35 (28.92%)	39 (32.23%)	47 (38.84%)	121

Delivery mode:				
Vaginal	17 (24.6%)	13 (18.8%)	39 (56.5%)	69
Cesarian section	18 (35.3%)	25 (49.0%)	8 (15.7%)	51

GA				
24–28 weeks	4 (44.4%)	4 (44.4%)	1 (11.1%)	9
29–32 weeks	8 (30.7%)	6 (23.1%)	12 (46.2%)	26
33–37 weeks	21 (33.8%)	19 (30.7%)	22 (35.5%)	62
>38 weeks	2 (8.3%)	10 (41.7%)	12 (50.0%)	24
Onset of sepsis^1^	18.3 (14.0) hours	13.0 (7.1) days		

1 For this variable we calculated the mean (with standard deviation). Notes: GA-gestational age., EOS-early onset sepsis, LOS-late onset sepsis.

A higher percentage of cesarean deliveries occurred in the LOS group (49%) compared to the EOS group (33.3%), when considering distribution across delivery modes. When analyzing estimated gestational age (EGA), the LOS group had a higher percentage of newborns after 38 weeks of gestation (41.7%) compared to the EOS group (8.3%). The rate of sepsis cases among preterm infants born between 24–28 weeks of gestation was almost the same (44.4%) in both groups. The onset of sepsis varied significantly between the EOS and LOS groups, with mean durations of 18.3 hours (~0.75 days) and 13 days, respectively.

There is a significant and noteworthy contrast observed in the percentage of patients with positive blood cultures between the distinct study groups, showcasing a substantial disparity. In the EOS group, the percentage stands at 51.43%, while in the LOS group, it significantly rises to an impressive 89.75%. The prevailing trend unveils that Klebsiella spp dominates as a prevalent microorganism in EOS cases, accounting for a striking 44.4%, closely followed by E. coli at 27.78%. Conversely, within LOS cases, Klebsiella spp maintains its dominance with a notable prevalence of 42.85%, whereas E. coli assumes a lesser role at 11.42%. Additionally, other significant microorganisms identified include Staphylococcus aureus, Serratia marcescens, and Enterobacter cloacae; although their occurrence is comparatively reduced in contrast to the primary microorganisms mentioned earlier. Further analysis of the data in Table 2 provides a detailed breakdown identifying the specific microorganisms responsible for each sepsis group examined.

**Table 2.** Descriptive statistics for patients with confirmed culture results.

**Variables**	**EOS**	**LOS**	**Overall**
Negative blood culture	17 (48.57%)	4 (10.25%)	21
Positive blood culture	18 (51.43%)	35 (89.75%)	53

Microorganism for BC+ (positive blood culture)			
Klebsiella spp	8 (44.4%)	15 (42.85%)	23
Escherichia coli	5 (27.78%)	4 (11.42%)	9
Staphyloccocus aureus	2 (11.12%)	2 (5.71%)	4
Enterobacter	1 (5.55%)	2 (5.71%)	3
Serratia marcescens	2 (11.12%)	6 (17.14%)	8
Enterococcus	0 (0%)	3 (8.57%)	3
Pseudomonas aeruginosa	0 (0%)	2 (5.71%)	2
Others	0 (0%)	1 (2.85%)	1

**Fig. 1.**
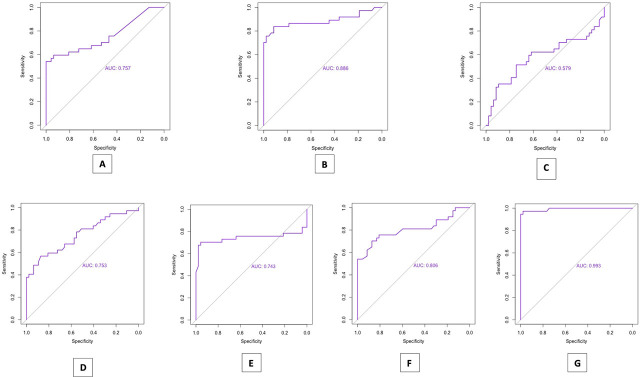
Comparative Diagnostic Performance of Biomarkers and laboratory findings in Participant Groups: Specificity and Sensitivity Analysis through ROC Curve Evaluations; A – ROC curve for C-reactive protein; B – ROC curve for procalcitonin; C – ROC curve for leukocytes; D – ROC curve for platelets; E – ROC curve for neutrophils; F – ROC curve for LDH; G – ROC curve for ferritin.

We performed a comparative analysis of various biomarkers and laboratory findings, such as CRP, PCT, leukocyte count, neutrophil count, platelets (TR), LDH, and ferritin levels in all participant groups. We attempted to identify the biomarker with the best diagnostic performance, assessed in terms of specificity and sensitivity. To aid in this, we utilized ROC curves. Figure 1 illustrates the specificity and sensitivity features of the analyzed biomarkers and parameters, obtained from the ROC curve analyses.

The first assessed biomarker was CRP. In Figure 1A, the ROC curve for this biomarker in relation to our study groups shows an area under the curve (AUC) of 0.757, indicating good discriminative ability. However, it is not perfect, with a sensitivity of 54.1% and a specificity of 100%. The cutoff value for this test was 5.12. These results indicate that while CRP can detect and exclude negative patients, it is not entirely reliable as it can only diagnose a little over half of the positive cases using this marker.

Figure 1B displays the ROC curve for PCT, producing an AUC of 0.886, indicating a high capability to differentiate between individuals with or without sepsis in different groups. Moreover, the test exhibits a specificity of 91.4% and a sensitivity of 83.7%, resulting in high accuracy in diagnosing the condition. The threshold for this marker is 0.79.

Leukocyte count was established as a parameter for diagnosing infection in all three groups. Figure 1C shows an AUC of 0.579, indicating a limited ability to distinguish between individuals with or without sepsis across various groups. The test has a specificity of 74.4% and a sensitivity of 51.3%, leading to poor accuracy in diagnosing the condition. The marker threshold is 18.350/mm^3^.

Figure 1D describes how platelets can be used as a laboratory finding for sepsis, with an AUC of 0.753, suggesting good discriminative ability, a sensitivity of 56.7% and a specificity of 87.2%. Although the test shows good specificity, there can be room for improvement. The cut off value was set at 168.000/mm^3^.

Neutrophils were also accounted as a strong indicator for neonatal sepsis. With an AUC of 0.743, specificity of 95.7% and sensitivity of 70.2%, as seen in Figure 1E, it shows great potential as an indicator for sepsis. The cut off value for this finding was 45.4*10^3^/mm^3^.

LDH was assessed as well (Figure 1F), showing similar abilities to PCT, but lower sensitivity (70.2%) and lower specificity (87.2%). The threshold was set at 652 IU/L.

The most impactful biomarker seen in our study and demonstrated by the ROC curve was ferritin, with an AUC of 0.993, as observed in Figure 1G, a sensitivity of 97.2% and specificity of 97.8%. The cut off value was 248.5ng/ml for this marker.

Table 3 shows the results of the Wilcoxon signed rank test, revealing a statistically significant difference between the initial (at admission) and final measures (at 72 hours after onset) of LDH and ferritin levels in both EOS and LOS groups. However, no significant differences were observed for those two variables within the control group.

**Table 3.** Comparing the first and last measured values for continuous variables using the two-sample Wilcoxon signed rank test. Presented are the p-values.

**Variables**	**EOS (p-value)**	**LOS (p-value)**	**Control (p-value)**
CRP	0.787	0.798	**0.029**
Procalcitonin	0.081	0.934	0.739
Leukocyte	0.740	0.214	**0.002**
Neutrophils	0.245	0.942	0.144
Platelets	0.342	0.364	**0.00001**
LDH	**0.006**	**0.00001**	0.061
Ferritin	**0.0002**	**0.0038**	0.529

In bold are statistically significant differences. Notes: CRP-C reactive protein, LDH-Lactate dehydrogenase, EOS-early onset sepsis, LOS-late onset sepsis.

Significant differences were noted between the initial and last measurements for CRP (p = 0.029), leukocyte count (p = 0.002), and platelets (p = 0.00001) for the control group. The significance of these markers in non-infected newborns essentially revolves around their roles in the neonate’s initial adaptation to postnatal life and the resolution of any non-infectious, birth-related inflammatory responses.

**Fig. 2.**
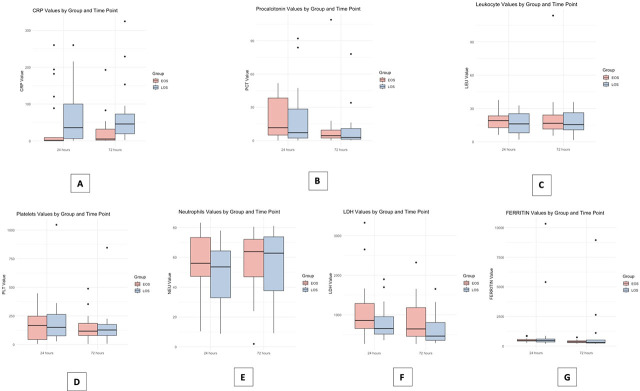
Comparative analysis of biomarker profiles in EOS and LOS groups at different time points (24 and 72 hours after onset); A – Boxplot for C-reactive protein; B – Boxplot for procalcitonin; C – Boxplot for leukocytes; D – Boxplot for platelets; E – Boxplot for neutrophils; F – Boxplot for LDH; G – Boxplot for ferritin.

We examined the median, distributions and outliers of the biomarkers and parameters used between the EOS and LOS groups, as well as the values measured during the 24 hours of infection compared to those at 72 hours after the disease onset. Figure 2 describes each biomarker accordingly.

The values of CRP at 24 hours for EOS show a high median and wide distribution, indicating greater variability and some high outliers. For LOS, the median level is near zero, with one notable high outlier, implying generally low CRP levels by 24 hours after the onset of the disease, with an exceptional high value. By 72 hours, EOS presents a slightly higher median than LOS and fewer outliers, indicating more consistent, but still variable CRP levels. These observations can be seen in Figure 2A.

Figure 2B describes procalcitonin measurements. The median PCT value for the EOS group at 24 hours is higher than the LOS group at the same time point. The data spread is considerable, with several outliers, indicating some very high PCT values. By 72 hours, the median PCT value for the EOS group has decreased, and the interquartile range has narrowed, suggesting reduced variability. No outliers are observed at this time point. The 72-hour levels for LOS appear to have stabilized as well.

Both EOS and LOS groups show similar ranges of leukocyte counts at both time points (Figure 2C), with medians that appear to be relatively stable from 24 to 72 hours. The EOS group has a slightly left-skewed distribution at both time points, with a single outlier at 72 hours, while LOS shows a more symmetric distribution with no outliers. This indicates that there are no dramatic changes in leukocyte count over time for both groups.

Figure 2D describes platelet values. Across both groups and time points, the median platelet count is well above the sepsis indicator threshold. However, the presence of outliers, particularly in the EOS group at 24 hours indicates individual cases of severe sepsis.

Neutrophil counts for both groups are generally within the same range at both 24 and 72 hours, with median counts slightly increasing at the later time point. The EOS group displays a more variability that the LOS group, but not significantly so (Figure 2E).

Figure 2F shows that both EOS and LOS groups present stable median LDH levels from 24 to 72 hours, and no significant changes over time for the majority of patients. The presence of outliers in both groups, particularly by 24 hours, suggests that there are newborns with markedly elevated enzyme levels. The levels for both groups do not show drastic changes over the observed time period, as indicated by the consistent median values.

Figure 2G shows that the median ferritin values were low at both time points. The existence of outliers exhibiting exceptionally elevated ferritin levels at 24 and 72 hours in both the EOS and LOS groups suggests the presence of noteworthy clinical consequences. The distribution remains relatively constant from 24 to 72 hours.

Comparing the two sepsis groups (EOS and LOS) and the control group 24 hours after onset, Table 4 shows that there is a significant difference in markers for the majority of variables. Nevertheless, it was found that leukocyte levels did not exhibit statistical differences when comparing EOS vs Control, LOS vs Control, and EOS vs LOS groups, respectively. When comparing the EOS and LOS study groups, a statistically significant difference in CRP levels was observed (p = 0.00001).

**Table 4.** Comparing the second values (at 24 hours after onset) using the two-sample Wilcoxon signed rank test. Presented are the p-values.

**Variables**	**EOS vs Control (p-value)**	**LOS vs Control (p-value)**	**EOS vs LOS (p-value)**
CRP	**0.0015**	**0.000005**	**0.00001**
Procalcitonin	**0.00005**	**0.00001**	0.741
Leukocyte	0.4655	0.254	0.926
Neutrophils	**0.00001**	**0.000001**	0.668
Platelets	**0.0076**	**0.0008**	0.956
LDH	**0.0009**	**0.0006**	0.130
Ferritin	**0.00001**	**0.000001**	0.063

**In bold are statistically significant differences.** Notes: CRP-C reactive protein, LDH-lactate dehydrogenase.

During the study, we also examined the connection between surgical interventions and variables related to mechanical ventilation in newborns with LOS, as shown in Table 5. These newborns were the only ones who underwent surgical interventions, which heightened the risk of sepsis development. The chi-square test was employed to statistically analyze the relationship between these categorical variables. Significant associations were observed between Surgical Intervention and SIMV variables (p=0.0344).

**Table 5.** Relationship among the categorical variables. The association between surgical intervention and the necessity of oxygen supplementation for LOS patients.

**Variable**		**Surgical interventions in LOS group (n=)**		**p-value**
		**No**	**Yes**	
Oxygen therapy	No	6	2	1
	Yes	22	9	

High flow nasal canula	No	21	10	0.397
	Yes	7	1	

nCPAP	No	27	9	0.189
	Yes	1	2	

nIPPV/nCPAP	No	27	11	1
	Yes	1	0	

SIMV	No	19	3	**0.0344**
	Yes	9	8	

In bold are statistically significant differences. Notes: nCPAP-nasal continuous positive airways pressure, nIPPV-Noninvasive positive pressure ventilation, SIMV-Synchronized intermittent Mandatory Ventillation.

## Discussion

The final diagnosis depended on the results obtained from the different bacterial cultures. Due to the inherent constraints of these cultures, such as the necessary waiting period and the following confirmation process, biomarkers are the only quick diagnostic method available for this specific group of vulnerable newborns. Relying on biomarkers for quick diagnosis highlights a significant deficiency in promptly managing sepsis in newborns. The extended period required for culture growth and confirmation can delay the start of specific therapeutic treatments, increasing the risk of negative outcomes in this group. Therefore, the quickness with which biomarkers can indicate the existence of sepsis is a crucial benefit. It enables the early identification and treatment of this life-threatening condition, reducing the related risks and enhancing the outlook for affected newborns. Our study highlights the importance of improving the precision and dependability of biomarker-based diagnostics to address the challenge of timely detection and the constraints of conventional culture methods.

While biomarkers such as C-reactive protein, procalcitonin, and laboratory findings such as leukocyte count, neutrophils, and platelets did not show statistically significant differences between the specified groups within the studied time frames (24 hours versus 72 hours after the onset of infection), it is important to highlight that lactate dehydrogenase and ferritin levels exhibited notable distinctions in both early-onset sepsis (EOS) and late-onset sepsis (LOS) categories. Comparisons across EOS, LOS, and Control groups highlighted CRP’s significant variation between EOS and LOS, emphasizing its potential as a discriminating factor between the onset types. However, the lack of significant differences in other biomarkers across these groups indicates the need for a more nuanced understanding of biomarker behavior in different sepsis sub-types.

These findings emphasize the potential value of these specific biomarkers and parameters in distinguishing between different stages of sepsis, providing insights into their diagnostic importance and clinical relevance. Some studies that evaluated C-reactive protein, along with the total number of leukocytes, as being a biomarker used to identify newborns with sepsis, found these are still useful for evaluating neonates with suspected infection [3]. Sharma D. et al. describes the role of CRP in monitoring the response to treatment of infected neonates, rather than using this marker as a diagnosis tool [29].

Identifying sepsis in clinical practice is challenging due to of its non-specific signs and early clinical manifestations. Our retrospective study aimed to evaluate biomarkers and their limitations in diagnosing neonatal sepsis across various onset categories: early-onset sepsis (EOS), late-onset sepsis (LOS), and a control group.

Kamial Z. et al revealed that the sensitivity, specificity, and best cut-off point for ferritin was statistically significant (p=0.507). The same is described for procalcitonin and CRP [25]. Our study reveals that the best sensitivity and specificity was represented by ferritin, followed by procalcitonin and LDH (AUC=0.99, 0.88, 0.80). The severity of infection was found to be correlated with both PCT and CRP levels at birth, as well as at 24 and 48 hours of life in the presence of sepsis, as stated by Chiesa C. et al. [30]. Furthermore, Gatseva P. et al. states that procalcitonin is commonly utilized for diagnosing sepsis and appears to be more sensitive than CRP in the initial phases of bacterial infections [31].

The ferritin and lactate dehydrogenase revealed promising results for both EOS and LOS groups, in both time frames (at 24 hours after onset and 72 hours, respectively). Research indicates that neonates with sepsis have elevated ferritin levels compared to healthy neonates [25]. Additionally, plasma LDH levels were significantly higher in individuals with early sepsis compared to those without, with statistical significance at p<0.01 [26]. A study conducted by Zein et al. concluded that elevated plasma LDH levels in severe infections, indicating tissue damage, were not a reliable predictor of mortality in severe sepsis patients after 48 hours [32].

Our study found that late onset sepsis (LOS) was almost as common as early onset sepsis (EOS) because we focused on the initial episode of sepsis in newborns with extended hospital stays, who are more vulnerable to hospital-acquired infections. Newborns who spend extended periods in the NICU are at a high risk of developing LOS [33]. A multicenter survey by Stoll et al. [34] indicated that 21% of very low birth weight (VLBW) infants experienced at least one episode of sepsis.

The changes in LDH and ferritin levels over time showed significant differences between the early-onset sepsis (EOS) and late-onset sepsis (LOS) groups, suggesting their potential as markers for disease progression. The unique patterns seen in the changes of markers between the sepsis and control groups confirm the usefulness of these markers in monitoring disease and determining prognosis. Ferritin, along with CRP can be used together in order to distinguish groups of neonates with sepsis who have different mortality risks and systemic inflammation responses [35].

The most reliable method for diagnosis remains culture of blood or cerebrospinal fluid, but it is time-consuming and may delay prompt treatment. Hence, dependable biomarkers are essential for initiating antibiotherapy and subsequently adjusting it based on culture results.

Furthermore, our study identified significant associations between specific clinical interventions, such as surgical interventions, and variables related to mechanical ventilation in neonatal sepsis cases. These associations could serve as important clinical indicators to help guide treatment strategies. Prolonged intubation and mechanical ventilation greatly increase the risk of systemic infection, particularly ventilation-associated pneumonia, which is commonly considered a nosocomial infection [25,36]. Diagnostic criteria encompass: temperature instability, alterations in blood gases, tachypnea, need of increased ventilation parameters, and a duration of mechanical ventilation exceeding 48 hours [35]. The incidence of late onset sepsis in hospitalized neonates ranges from 0.6% to 14%, based on literature [15]. Risk factors for LOS include prematurity, a prolonged exposure to invasive procedures, delayed enteral feeding, surgical intervention and underlying respiratory and cardiac disease.

The limitations of our study included its retrospective design and small sample size. Future studies could enhance the strength of our results by exploring additional biomarkers such as proinflammatory cytokines like Il-6 or tumoral necrosis factor, using larger study groups and adopting a prospective study design. Moreover, exploring new biomarkers such as presepsin or endocan, or combining multiple biomarker panels could enhance diagnostic accuracy and help differentiate between various sepsis subtypes.

In the future, we plan to introduce a neonatal sepsis prediction score to identify newborns at risk more accurately and reduce unnecessary antibiotic use. Various predictor scores for sepsis are available, and many of them evaluate temperature, platelets, gastric residuals, hypo- or hyperglycemia or other laboratory findings [37]. Some of these scores consisted exclusively of clinical variables [38], while others used many categories of variables such as the HeRO score. Endothelial cell-specific molecule-1 named Endocan – is a circulating 50-kDa dermatan sulphate proteoglycan expressed by endothelial cells [33]. Serum concentration of endocan is elevated in patients with sepsis and its level is correlated with disease severity [9].

Enhancing medical care for pregnant women, identifying premature birth risks, and detecting neonatal sepsis are vital. Utilizing prediction scores can anticipate sepsis, leading to better outcomes.

In our previous study, we determined that ferritin and lactate dehydrogenase serve as biomarkers for sepsis. However, we now aimed to investigate whether these biomarkers are more effective than the commonly used biomarkers such as C-reactive protein, procalcitonin, neutrophils, leukocytes, and platelets.

Despite our limitations, the study provides insights into the complexities of diagnosing neonatal sepsis and the challenges associated with relying solely on conventional biomarkers. The varying performance of different biomarkers across different onset groups emphasizes the need for a multifaceted approach to diagnosis. This approach should incorporate clinical assessments, imaging studies, and evolving biomarker panels to improve diagnostic precision and inform targeted therapeutic interventions

## Conclusions

Traditional biomarkers such as CRP, PCT, leukocyte count, neutrophils, and platelets have diagnostic limitations regarding neonatal sepsis. Our study highlights how LDH and ferritin can enhance diagnostic accuracy for both EOS and LOS. Ferritin specifically demonstrated high sensitivity and specificity for early sepsis detection. Integrating these innovative biomarkers with traditional ones could create a more reliable diagnostic system, reducing the need for culture methods and enabling timely interventions. This strategy could improve the diagnosis and treatment of neonatal sepsis, boosting survival rates. Future research should continue to verify these findings with additional bio-markers and larger studies to enhance clinical practice.
